# Traditional Chinese Medicine Containing Arsenic Treated MDS Patients Effectively through Regulating Aberrant Hypomethylation

**DOI:** 10.1155/2020/7469809

**Published:** 2020-03-07

**Authors:** Qing-bing Zhou, Qian-zhe Zhu, Hong-zhi Wang, De-xiu Wang, Zheng-tang Liu, Yong-gang Xu, Xiao-mei Hu, Rou Ma, Feng-qin Xu

**Affiliations:** ^1^Institute of Geriatric Medicine, Xiyuan Hospital, China Academy of Chinese Medical Sciences, Beijing 100091, China; ^2^Department of Hematology, Xiyuan Hospital, China Academy of Chinese Medical Sciences, Beijing 100091, China

## Abstract

Aberrant hypermethylation and hypomethylation both play important roles in myelodysplastic syndrome (MDS). Hypomethylating agents targeting hypermethylation have been employed for the MDS treatment, but the treatment effect is limited. Novel drugs for DNA hypomethylation-targeted therapy may be needed to improve clinic efficacy for the treatment of MDS. Chinese medicine (CM) herbs have been used to treat MDS for many years in our hospital. However, the long-term treatment effect and mechanism remain unclear. In this study, all 135 patients received CM treatment for at least 36 months. The response rates for CM treatment were 81.53% (106/130) for hematological improvement in 130 MDS-RCMD patients and 80% (4/5) for bone marrow CR in 5 MDS-RAEB patients, respectively. The Human Methylation 850K BeadChip showed that 115 genes (50.88%) were aberrantly hypomethylated in 5 MDS patients compared with 3 healthy individuals. GO-analysis showed that these hypomethylated genes participated in many cancer-related biological functions and pathways. Furthermore, 60 genes were hypermethylated and the protein expression level of DNMT1 was significantly increased in the 5 MDS patients after 6 months of CM treatment. Our study suggests that CM can improve aberrant hypomethylation by increasing DNMT1 expression in MDS. The data support the clinical application of CM herbs containing arsenic as an innovative hypermethylation-inducing regimen for the treatment of MDS.

## 1. Introduction

Myelodysplastic syndromes (MDS) are a group of myeloid clonal diseases that originate in hematopoietic stem cells and are characterized by ineffective hematopoiesis, refractory hematopoiesis, hematopoietic failure, and a high risk of transformation to acute myeloid leukemia (AML) [1]. Although many therapeutic strategies have been employed, the disease remains incurable [2, 3]. The pathophysiology of MDS involves epigenetic, genetic, and cytogenetic aberrations [4]. Aberrant DNA methylation plays a key role in MDS. Abnormal DNA hypermethylation has elicited great interest because of its direct impact on tumor suppressor genes. The introduction of hypomethylation agents (HMAs) approved for MDS represents the most significant example of this progress [5]. Clinical studies have shown that the clinical effective rate of HMAs including azacitidine (AZA) and decitabine (DAC) is approximately 40% in higher-risk MDS patients; HMA treatment failure is often observed and associated with a median survival time of less than 5 months [6]. Thus, novel drugs for DNA methylation-targeted therapy are needed to improve the clinical efficacy of the treatments for MDS.

Cancer is also related to aberrant DNA hypomethylation, which affects numerous genomic regions and drives the evolution of leukemia in MDS [7]. DNA hypomethylation plays a crucial role in cancer because it results in the transcriptional activation of oncogenes. For example, aberrant hypomethylation of the protooncogenes c-myc and c-fos has been found in MDS and AML patients [8]. The frequency of SALL4 hypomethylation is significantly increased in patients with higher-risk MDS and the hypomethylation of Let-7a-3 is associated with a poor prognosis in MDS patients [9, 10]. Therefore, a drug targeting DNA hypomethylation may be useful for the treatment of MDS patients. However, there are no such hypermethylation agents at present.

Chinese medicine (CM) is characterized by a special theory and the application history in China is more than 3,000 years. In our hospital, the CM herbs are composed of Qinghuang Powder (containing As_2_S_2_) and Bupi Yishen Decoction, which have been used to treat patients with MDS for more than 30 years. Our previous study indicated that CM treatment was effective in MDS patients [11]. However, that study was mainly based on short-term clinical observation, and the long-term clinical efficacy and treatment mechanism are still unclear.

Considering the importance of DNA methylation in MDS, we assumed that DNA methylation may be the target of the CM formulation. The following experimental protocols were used to prove this hypothesis. First, we retrospectively analyzed the data from 135 MDS patients who received CM treatment for more than 3 years. Subsequently, methylation changes in 5 MDS patients who received CM treatment were examined after treatment by an Illumina Human Methylation 850K array. Bone marrow from 3 healthy donors was obtained as a control. Finally, Western blotting was used to observe the protein expression of DNA methyltransferases (DNMT1, DNMT3a, and DNMT3b) in 5 MDS patients after the CM treatment.

## 2. Materials and Methods

### 2.1. Patients

The clinical efficacy of CM treatment in MDS patients was analyzed retrospectively in this study. MDS patients were recruited from the Xiyuan Hospital between January 2008 and March 2018, and the diagnostic criteria for MDS were the 2008 WHO classification system [12]. All patients were treated with CM for at least 36 months and 135 MDS patients were included. This research was approved by the Xiyuan Ethical Committees (No. 2018XLA005-2).

### 2.2. Treatments

Xiyuan Hospital provided all the herbs used in this study. QHP capsule (containing 0.1 g realgar and 0.2 g indigo) was administered at a dose of 0.3 g/d; a soup medicine, consisting of Rhizoma Dioscoreae (15 g), Radix Rehmanniae (20 g), Fructus Corni (20 g), Cortex Moutan (10 g), Poria (15 g), Fructus Psoraleae (15 g), Semen Cuscutae (20 g), Rhizoma Atractylodis Macrocephalae (20 g), Radix Pseudostellariae (30 g), Rhizoma Alismatis (20 g), Radix Polygoni Multiflori (25 g), Zingiberis Recens Rhizoma (15 g), Fructus Jujubae (15 g), Radix Morindae Officinalis (15 g), and Ramulus Cinnamomi (15 g) was administered orally twice a day. The clinical efficacy was determined according to the response criteria, established by the International Working Group [13, 14].

### 2.3. Human Methylation 850K BeadChip Analysis

Five MDS patients were treated with CM for 6 months and bone marrow samples were collected before and after treatment. Three healthy donors were used as controls. DNA was extracted from the bone marrow samples, and methylation was evaluated by 850K DNA methylation array, which is a device for DNA methylation detection with highly reproducible results [15]. In our study, the methylation status in the DNA samples was analyzed by the Illumina Human Methylation 850K array. Briefly, an EZ DNA methylation kit (Zymo Research, CA, USA) was used and the bisulfite conversion of 1 *μ*g DNA was performed for each sample. Then, the bisulfite-treated DNA was hybridized on Human Methylation 850K BeadChip, following the Infinium HD Methylation protocol. The SQ fluorescence scanner was used. Fully methylated DNA produces a ratio that approaches 1, whereas if methylation is completely absent, then the ratio would approache 0. Differentially methylated genes were analyzed by GO-analysis, which is an important bioinformatics tool for screening related functions [16, 17].

### 2.4. Methylation Validation by Pyrosequencing

Validation of methylation data was performed in a different sample set composed of 5 samples from MDS patients before treatment and 3 from healthy donors using pyrosequencing. The primers were designed using PSQ assay design software. The primers sequences of 2 genes were listed in [Supplementary-material supplementary-material-1]. Hot start high-fidelity Taq DNA polymerase (Qiagen, Germany) was used to perform the PCR reaction. The amplification conditions were 98°C for 10 seconds, followed by 55°C for 30 seconds, and 72°C for 30 seconds for 35 cycles. The specific PCR products were then subjected to quantitative pyrosequencing analysis using a PyroMark Q96 system (Qiagen, Germany) according to the manufacturer's instructions. The results were analyzed by Pyro Q-CpG software.

### 2.5. Western Blotting

We chose 5 MDS patients for Western blotting. These patients were treated with CM for 6 months and bone marrow samples were collected before and after treatment. RBC Lysis Buffer was used to eliminate red blood cells in the bone marrow samples. Protein was extracted with the use of Protein Extraction Kit (Gene pool, China) according to the manufacturer's instructions. Then the protein concentrations were measured by a BCA protein assay. Next, the protein samples were separated by SDS-PAGE and transferred to a PVDF membrane. After blocking in 5.0% nonfat milk for 1 h at room temperature, the specific primary antibodies were incubated with the PVDF membrane at 4°C overnight. Then secondary antibodies were added, and enhanced chemiluminescence (ECL) reagents (Thermo, USA) were used to detect the antigen-antibody binding. Quantity One v.4.6.2 was utilized for the quantification of the total gray area of each protein band.

### 2.6. Statistical Analysis

Bayesian and linear regression approaches were employed to evaluate the differences between the values of the methylation status for bone marrow samples that were determined by the Human Methylation 850K BeadChip. The significant criteria for the values of the methylation status of CpG sites was a *p* value <0.05 and a minimum change of ±0.1 in the *β*-values. The change in the expression of proteins in the MDS patients was analyzed with a paired *t*-test. *p* < 0.05 was considered significant.

## 3. Results

### 3.1. Patient Characteristics

A total of 135 patients (130 RCMD and 5 RAEB) were included in the final analysis. Clinical characteristics are listed in Table 1. Overall, the median age was 37 years old, 50.38% of the patients were female, and 5.93% and 94.07% of the patients were IPSS intermediate-1 and intermediate-2, respectively. In total, 72.7% of the patients had a normal karyotype among these 135 patients. The number of patients with bilineage or trilineage cytopenia was 112 (82.96%).

### 3.2. Response to CM

The clinical efficacy of CM in MDS patients was analyzed retrospectively. All 135 MDS patients (including 5 RAEB patients and 130 RCMD patients) received CM treatment for at least 36 months and the median treatment time was 60 months. The results showed that 80 of 103 anemic patients (77.67%; 95% CI: 69.63%–85.71%) showed a hematologic improvement-erythroid (HI-E) response. Among the neutropenic patients, 48 of 91 achieved a hematologic improvement-neutrophil (HI-N) response (52.75%; 95% CI: 42.5%–63.00%). In total, 62 of 115 (53.91%; 95% CI: 44.80%–63.02%) thrombocytopenic patients achieved a hematologic improvement-PLT (HI–P) response. Hematological improvement (HI-E or HI-N or HI-P) was achieved in 106 of 135 patients (78.52%; 95% CI: 71.59%–85.45%) (Table 2). The response in the RAEB patients (*n* = 5) was classified as mCR in 4 patients and failure in 1 patient (Table 3).

### 3.3. Many Abnormally Hypomethylated Genes Existed in MDS Patients

**Figure 1:**
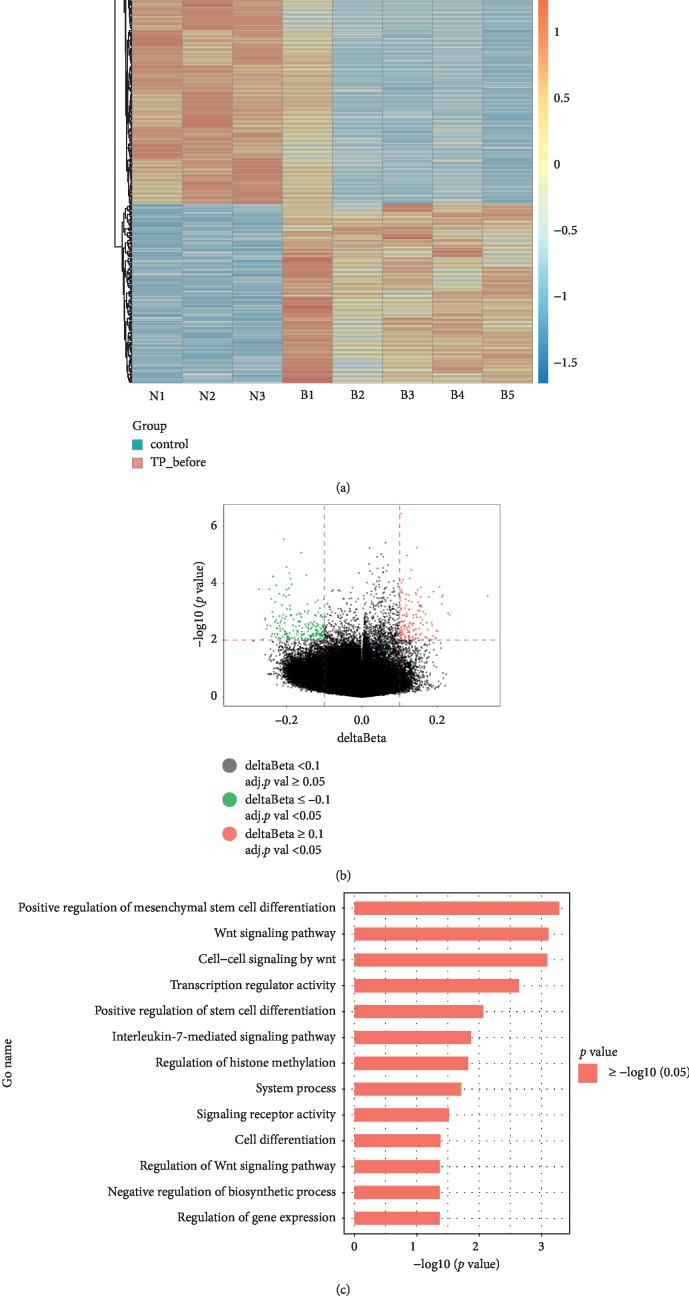
Differential methylation study of bone marrow samples from 5 MDS patients before treatment versus those from 3 healthy individuals. (a) Heatmap representing a supervised cluster centered on the median of the methylation levels between 5 MDS patients before treatment (TP_before) and 3 healthy individuals. Samples are represented as TP_before (salmon orange) and control (turquoise). Hypermethylated site probes in MDS patients are represented in orange, and hypomethylated probes are represented in blue. (b) Volcano plot representation of the methylation of significant gene sites. Hypomethylated probes are represented in green colour, and hypermethylated probes are represented in red. Red lines delineate ±0.1 methylation differences between the MDS patients and healthy donors and the dotted line represents a *p* value threshold of 0.05. (c) Significantly changed GO terms for hypomethylated genes in the MDS patients. The *y* axis shows the category and the *x* axis shows the –LgP. The larger –LgP indicated a smaller *p* value.

There was no difference in age between MDS patients and donors (*p* value = 0.88). Then we analyzed data from >853,000 sites in BM samples from 5 MDS patients before treatment and 3 healthy individuals with the Illumina Methylation EPIC BeadChip (Tables [Supplementary-material supplementary-material-1] and [Supplementary-material supplementary-material-1]). Bayesian and linear regression tests showed 336 sites that were significantly differentially methylated between the MDS patients and healthy donors (*p* value <0.05 and methylation differences ±0.1). Among the sites, 198 sites were hypomethylated and 138 were hypermethylated in the MDS patients, which corresponded to 115 hypomethylated and 111 hypermethylated genes, respectively (data not shown). A heatmap and volcano plot comparing the 5 MDS patients with the 3 healthy individuals are shown in Figures 1(a) and 1(b). On the basis of GO-analysis, the 115 hypomethylated genes participated in many cancer-related functions and pathways including cell differentiation, Wnt receptor signaling, and signaling receptor activity and so on (Figure 1(c)).

### 3.4. Validation of Hypomethylated Genes in MDS Patients before Treatment

**Figure 2:**
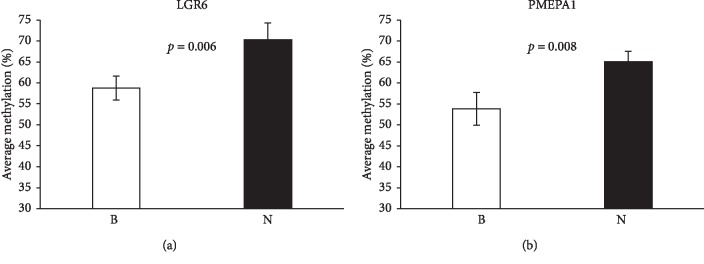
Methylation analysis by direct bisulfite pyrosequencing in MDS patients before treatment. 2 genes were chosen for the validation. Mean methylation value for each CpG analyzed by direct bisulfite pyrosequencing in DNA from bone marrows. The mean methylation values for LGR6 and PMEPA1 in MDS patients B were listed and healthy donors N were added as a control. As shown in Figures (a) and (b), LGR6 (*p* value = 0.006) and PMEPA1 (*p* value = 0.008) were hypomethylated in MDS patients.

We analyzed the methylation status of LGR6 and PMEPA1 by pyrosequencing. These 2 genes were significantly hypomethylated in MDS patients (*p* value <0.05) compared with those in healthy donors (Figure 2). LGR6 (*p* value = 0.006) and PMEPA1 (*p* value = 0.008) were hypomethylated in patients, which were consistent with the results from the Human methylation 850K.

### 3.5. CM Treatment Improved Hypomethylation in MDS Patients

**Figure 3:**
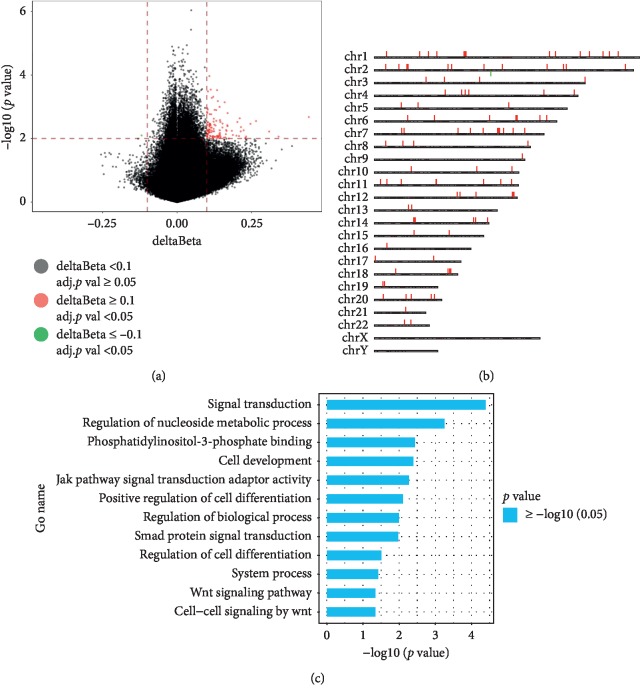
Changes in the DNA methylation status in 5 MDS patients after treatment by the Human Methylation 850K BeadChip. (a) Volcano plot representation of the methylation of significant gene sites. Hypomethylated probes are represented in green colour and hypermethylated probes are represented in red. Red lines delineate ±0.1 methylation differences between the MDS patients after treatment and the MDS patients before treatment, and the dotted line represents a *p* value threshold of 0.05. (b) Distribution of differentially methylated sites in the chromosomes of the MDS patients: red represents hypermethylated sites and green represents hypomethylated sites. (c) Significantly changed GO terms for hypermethylated genes induced by CM treatment. The *y* axis shows category, and the *x* axis shows –LgP. The larger –LgP indicated a smaller *p* value.

Five MDS patients were treated with CM for 6 months, and bone marrow samples were collected before and after treatment ([Supplementary-material supplementary-material-1]). Then, we conducted an analysis of the changes in DNA methylation status observed in the 5 MDS patients after CM treatment using the Infinium Human Methylation 850K BeadChip. B1, B2, B3, B4, and B5 and A1, A2, A3, A4, and A5 represent the patients before and after treatment, respectively. In total, 109 sites in the MDS patients were significantly changed after the CM treatment, which corresponded to 61 genes. Among the 61 genes, 60 genes were hypermethylated and only 1 gene was demethylated (data not shown). In Figures 3(a) and 3(b), red represents hypermethylated sites and green represents hypomethylated sites. The distribution of differentially methylated chromosomal sites in the patients before and after treatments is listed in Figure 3(b). GO-analysis showed that the genes with hypermethylation induced by CM treatment were related to signal transduction, cell development, Wnt signaling pathways, signaling receptor activity, and so forth (Figure 3(c)).

### 3.6. CM Treatment Increased the Protein Expression of DNMT1 in MDS Patients

**Figure 4:**
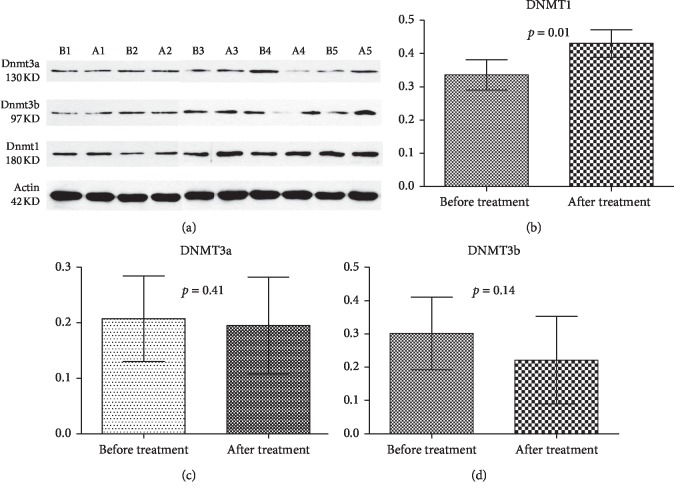
CM treatment increased the protein expression of DNA methyltransferase 1 in 5 MDS patients after treatment. (a) Five MDS patients were treated with CM for 6 months, and Western blotting was used to evaluate changes in the protein levels of DNMT1, DNMT3a, and DNMT3b. B1, B2, B3, B4, and B5 and A1, A2, A3, A4, and A5 represent patients before treatment and after treatment respectively. Gray values of DNMT1 (b), DNMT3a (c), and DNMT3b (d) were shown. *p* < 0.05 was considered significant.

We next focused on changes in the protein expression of DNA methyltransferases in 5 MDS patients. As shown in Figure 4, treatment could increase the expression of DNMT1 significantly (*p* value = 0.01), but had no effect on the expression of DNMT3a or DNMT3b (*p* value = 0.41 and *p* value = 0.14, respectively).

## 4. Discussion

To the best of our knowledge, the present work is the first to address the effects of CM treatment containing arsenic on DNA methyltransferases in MDS patients, demonstrating that this treatment could effectively treat MDS and improve aberrant hypomethylation in MDS patients by increasing DNA methyltransferase 1 expression. Our study indicated that CM containing arsenic may be an innovative hypermethylating agent for targeting DNA hypomethylation, which is distinct from hypomethylating agents.

The treatment choices for MDS patients mainly include hypomethylating therapy, chemotherapy, and immunotherapy. Azacitidine and decitabine represent the greatest progress in MDS treatment over the past decade. The response rate is approximately 50% in treated patients; however, the duration of response is transient, and HMA-treated MDS patients eventually lose responsiveness. For MDS patients with HMA treatment failure, standard therapeutic opportunities are few [18]. Our study showed that CM treatment may be a good choice for MDS patients. Consistent with our previous study, this study showed an 82.22% response rate in MDS patients [11]. The response rates to treatment were 81.53% (106/130) for hematological improvement in MDS-RCMD patients and 80% (4/5) for bone marrow CR in MDS-RAEB patients, respectively. Interestingly, anemic patients had a better response rate than neutropenic or thrombocytopenic patients (67.69% versus 37.69% or 47.69%, respectively), implying that CM treatment achieved its greatest therapeutic effect on the erythroid lineage. The prognosis for MDS-RAEB patients is very poor. In our study, 5 MDS-RAEB patients were enrolled for clinical observation. Their treatment times ranged from 36 months to 71 months. Four of the 5 patients achieved bone marrow CR, which indicated that this CM treatment may have a powerful effect on MDS-RAEB patients. In the future, double-blinded, randomized clinical trials are highly needed to confirm these results observed in the current study.

We have used CM herbs to treat patients with MDS for many years in Xiyuan Hospital. However, the mechanism underlying this effect remains unclear. A large number of papers have proven that DNA methylation plays a key role in MDS [19]. Hypomethylating agents targeting hypermethylation are already applied for MDS patients treatment, but the treatment effect is limited. In addition to hypermethylation, hypomethylation has also been observed in MDS patients. Hypomethylation can result in the transcriptional activation of oncogenes in cancer including MDS [20–23]. In our study, we also found that many abnormally hypomethylated genes were present in MDS patients compared with healthy donors, which is consistent with previous reports [24]. GO-analysis showed that these abnormally hypomethylated genes participated in cancer-related functions and pathways, which supported the importance of hypomethylation in MDS. In order to normalize the aberrant hypomethylation in MDS, drugs targeting hypomethylation are required.

In this study, we performed genome-wide methylation analysis of MDS patients before and after treatment with the Illumina Human Methylation 850K Array. The data showed that CM treatment induced DNA hypermethylation in MDS patients, which was an unexpected finding. Sixty genes were hypermethylated and only 1 gene was hypomethylated in response to CM treatment, and some of these genes deserved further study in the future. For example, CM treatment induced meningioma 1 (MN1) gene hypermethylation. The MN1 gene encodes a protein of 136-kDa, which is unique because it does not show homology to any known proteins [24]. Previous studies have demonstrated that MN1 is a potent oncogene involved in hematopoiesis that promotes self-renewal and proliferation and also blocks differentiation [25]. A clinical study showed that elevated MN1 expression in AML patients was a marker, associated with a worse prognosis [26]. On the other hand, GO-analysis of the 60 hypermethylated genes showed that these genes also participated in cancer-related functions and pathways. Taken together, these observations suggest that CM treatment led to the methylation of the cancer-related genes, which might be the main mechanism of action of CM in the treatment of MDS.

It is known that DNA methylation, or the covalent addition of a methyl group from *S*-adenosylmethionine (Sam) to cytosine mediated by DNMTs, is an essential epigenetic modification of the genome in mammalian cells [27]. At present, people found five DNMTs (DNMT1, DNMT2, DNMT3a, DNMT3b, and DNMT3L) that have been identified in mammals; however, only DNMT1, DNMT3a, and DNMT3b have methyltransferase activity [28]. In this study, changes in the protein expression of DNA methyltransferases in MDS patients were checked and an increase in the DNMT1 protein level was observed after the CM treatment. Meanwhile, the expression of DNMT3a and DNMT3b remained unaffected. These results showed us that CM treatment induced gene hypermethylation by increasing DNA methyltransferase1 expression, and this mechanism was different from that of the DNA methylation inhibitors decitabine and azacitidine [29].

In summary, our study suggests that CM treats MDS patients effectively. Furthermore, CM treatment can regulate aberrant hypomethylation by increasing the expression of DNMT1, which may be an innovative method of inducing hypermethylation. Whether these treatment effects will translate into better survival rates for MDS patients remains to be determined in clinical trials in the future.

## Figures and Tables

**Table 1:** Patients demographics and MDS disease characteristics.

Item	Case (%)
Age	
≥60 years	19 (14.07)
<60 years	116 (85.92)

Male	67 (49.62)

Female	68 (50.38)

WHO subtypes	
RCMD	130 (96.29)
RAEB-1	3 (2.22)
RARB-2	2 (1.49)

IPSS classification	
Intermediate-1 risk	8 (5.93)
Intermediate-2 risk	127 (94.07)

Cytogenetic findings	
Normal/diploid	93 (68.88)
+8	8 (5.92)
Complex (≥3 abnormalities)	5 (3.70)
20q-	5 (3.70)
-Y	3 (2.25)
Other	21 (15.55)

Cell-lineage cytopenia	
Unilineage	23 (17.04)
Bilineage	43 (31.85)
Trilineage	69 (51.11)

Baseline cytopenia(s)	
Anemia	6 (4.44)
Neutropenia	3 (2.22)
Thrombocytopenia	14 (10.37)
Anemia + neutropenia	11 (8.15)
Neutropenia + thrombocytopenia	10 (7.41)
Anemia + thrombocytopenia	22 (16.30)
Anemia + neutropenia + thrombocytopenia	69 (51.11)

RCMD: Refractory cytopenia with multilineage dysplasia; RAEB-1: Refractory anemia with excess blasts-1; RAEB-2: Refractory anemia with excess blasts-2.

**Table 2:** Hematological improvements induced by CM treatment in 130 MDS-RCMD patients.

Parameter	Case (%)	95% CI
HI-E (*n* = 103)	80 (77.67)	69.63–85.71
HI-N (*n* = 91)	48 (52.75)	42.5–63.00
HI-P (*n* = 115)	62 (53.91)	44.80–63.02
Bilineage (HI-E and HI-N, *n* = 74)	37 (50.00)	38.60–61.39
Bilineage (HI-N and HI-P, *n* = 74)	27 (36.49)	25.52–47.45
Bilineage (HI-P and HI-E, *n* = 84)	46 (54.76)	44.12–65.40
Trilineage (HI-E, HI-N and HI-P, *n* = 66)	26 (39.39)	27.60–51.18
HI-E or HI-N or HI-P (*n* = 135)	106 (78.52)	71.59–85.45

**Table 3:** Effect of CM treatment on 5 MDS-RAEB patients.

Patient	Diagnosis	Treatment time (months)	Efficacy
Patient 1	RAEB-2	71	Bone marrow CR

Patient 2	RAEB-1	45	Bone marrow CR

Patient 3	RAEB-1	36	Bone marrow CR

Patient 4	RAEB-1	38	Failure

Patient 5	RAEB-2	36	Bone marrow CR

## Data Availability

The data used to support the findings of this study are available from the corresponding author upon request.

## Supplementary Materials

Supplementary Materials Table S1: details of the primers sequences used in this study. Table S2: details of 5 MDS patients evaluated by the methylation 850K Beadchip. Table S3: details of 3 healthy donors evaluated by the Human Methylation 850K BeadChip.
