# Supplementary material for: Traditional Chinese Medicine Containing Arsenic Treated MDS Patients Effectively through Regulating Aberrant Hypomethylation
Source: Evid Based Complement Alternat Med. 2020 Mar 7;2020:7469809. doi: 10.1155/2020/7469809 (PMC7085376; doi:10.1155/2020/7469809)
Supplement: Supplementary Materials — Table S1: details of the primers sequences used in this study. Table S2: details of 5 MDS patients evaluated by the methylation 850K Beadchip. Table S3: details of 3 healthy donors evaluated by the Human Methylation 850K BeadChip. [file 7469809.f1.zip › 7469809.f1/table S1.docx]

**LGR6**

| \| Primer Set 1 \| Score: 94 \| \| --- \| --- \| \| General Warnings \| \|  \| \| --- \| \| | \| 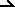 \| F1 \| GTTATTTTAGGGGTTTTGGTTGT \| \| --- \| --- \| --- \| \| 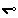 \| R1 \| CTTCCTCCCTCCCCTATATTAT \| \| 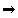 \| S1 \| GGGGTTTTGGTTGTTAT \| |
| --- | --- | --- | --- | --- | --- | --- | --- | --- | --- | --- | --- | --- | --- | --- | --- |

|  | PCR Product | Forward PCR Primer, F1 | Reverse PCR Primer, R1 | Sequencing Primer, S1 |
| --- | --- | --- | --- | --- |
| Length, nt | 86 | 23 | 22 | 17 |
| Position, 5'- 3' |  | 33 - 55 | 118 - 97 | 42 - 58 |
| Warnings | \|  \| \| --- \| | \|  \| \| --- \| | \|  \| \| --- \| | \|  \| \| --- \| |
| Tm, ºC |  | 58.8 | 58.4 | 47.0 |
| %GC | 31.4 | 34.8 | 45.5 | 41.2 |
| Sequence to Analyze | TTYGTTTTAG GGTTTGATTG GTAGTTTTA | | | |

**PMEPA1**

| \| Primer Set 1 \| Score: 99 \| \| --- \| --- \| \| General Warnings \| \|  \| \| --- \| \| | \| 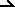 \| F1 \| GGGATTGTGGTTAATAGGTTTTTTAGTTGA \| \| --- \| --- \| --- \| \| 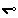 \| R1 \| ACACTAAATATAACCTCCCCACTT \| \| 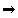 \| S1 \| ATTTGGTTTAGTTTAGGGA \| |
| --- | --- | --- | --- | --- | --- | --- | --- | --- | --- | --- | --- | --- | --- | --- | --- |

|  | PCR Product | Forward PCR Primer, F1 | Reverse PCR Primer, R1 | Sequencing Primer, S1 |
| --- | --- | --- | --- | --- |
| Length, nt | 107 | 30 | 24 | 19 |
| Position, 5'- 3' |  | 6 - 35 | 112 - 89 | 35 - 53 |
| Warnings | \|  \| \| --- \| | \|  \| \| --- \| | \|  \| \| --- \| | \|  \| \| --- \| |
| Tm, ºC |  | 62.4 | 61.4 | 45.7 |
| %GC | 30.8 | 33.3 | 37.5 | 31.6 |
| Sequence to Analyze | TTTTGGGYGT TATTATTAAT TTTAGG | | | |
